# Association between ambient and household air pollution with carotid intima-media thickness in peri-urban South India: CHAI-Project

**DOI:** 10.1093/ije/dyz208

**Published:** 2019-10-11

**Authors:** Otavio T Ranzani, Carles Milà, Margaux Sanchez, Santhi Bhogadi, Bharati Kulkarni, Kalpana Balakrishnan, Sankar Sambandam, Jordi Sunyer, Julian D Marshall, Sanjay Kinra, Cathryn Tonne

**Affiliations:** 1 Barcelona Institute for Global Health, Universitat Pompeu Fabra, CIBER Epidemiología y Salud Pública, Barcelona, Spain; 2 Public Health Foundation of India, New Delhi, India; 3 National Institute of Nutrition, Indian Council of Medical Research, Hyderabad, India; 4 Department of Environmental Health Engineering, Sri Ramachandra University (SRU), Chennai, India; 5 Department of Civil and Environmental Engineering, University of Washington, Seattle, WA, USA; 6 Department of Non-communicable Disease Epidemiology, London School of Hygiene and Tropical Medicine, London, UK

**Keywords:** Cardiovascular, air pollution, India, atherosclerosis, particulate matter

## Abstract

**Background:**

Evidence linking ambient air pollution with atherosclerosis is lacking from low- and middle-income countries. Additionally, evidence regarding the association between household air pollution and atherosclerosis is limited. We evaluated the association between ambient fine particulate matter [particulate matter with an aerodynamic diameter of ≤2.5 µm (PM_2.5_)] and biomass fuel use on carotid intima-media thickness (CIMT), a surrogate of atherosclerosis, in India.

**Methods:**

We analysed the third follow-up of the Andhra Pradesh Children and Parent Study cohort (2010–2012), which recruited participants from 28 peri-urban villages. Our primary outcome was mean CIMT, measured using a standardized protocol. We estimated annual average PM_2.5_ outdoors at residence using land-use regression. Biomass cooking fuel was self-reported. We fitted a within-between linear-mixed model adjusting for potential confounders.

**Results:**

Among 3278 participants (48% women, mean age 38 years), mean PM_2.5_ was 32.7 [range 24.4–38.2] µg/m^3^, and 60% used biomass. After confounder adjustment, we observed positive associations between within-village variation in PM_2.5_ and CIMT in all participants [1.79%, 95% confidence interval (CI), −0.31 to 3.90 per 1  µg/m^3^ of PM_2.5_] and in men (2.98%, 95% CI, 0.23–5.72, per 1  µg/m^3^ of PM_2.5_). Use of biomass cooking fuel was associated with CIMT in all participants (1.60%, 95% CI, −0.46 to 3.65), especially in women with an unvented stove (6.14%, 95% CI, 1.40–10.89). The point-estimate for the PM_2.5_ association was larger in sub-groups with higher cardiometabolic risk profile.

**Conclusions:**

Ambient and household air pollution were positively associated with CIMT in a peri-urban population of India, although with limited precision for some estimates. We observed differences in the association between ambient and household air pollution and CIMT by gender.

**Key Messages**

- To our knowledge, there is no previous evidence about the association between outdoor ambient fine particulate matter (PM_2.5_) and carotid intima-media thickness from populations in low- and-middle income countries.
- In this population-based study of 3372 participants, with annual mean ambient PM_2.5_ of 32.7 (range 24.4–38.2) µg/m^3^, annual mean PM_2.5_ was associated with carotid intima-media thickness among men.
- 60% of participants used biomass cooking fuel, which was strongly associated with carotid intima-media thickness in women cooking with an unvented stove.
- Women had higher values of carotid intima-media thickness compared with men, which might be attributed to high cumulative exposure to household air pollution.

## Introduction

Cardiovascular diseases are the leading cause of mortality and morbidity worldwide, including in many low- and middle-income countries (LMICs).^1^ India has experienced a rapid epidemiological transition, resulting in notable prevalence of hypertension, diabetes and obesity.^1^^,^^2^ India is also affected by high levels of ambient and household air pollution (HAP), resulting in a setting with high baseline cardiovascular risk and widespread exposure to high levels of air pollution.^1^^,^^2^

Long-term exposure to ambient particulate matter (PM) has been associated with risk of acute myocardial infarction, stroke and cardiovascular mortality.^3–5^ The most plausible pathway by which PM causes cardiovascular diseases is by promoting inflammation and atherosclerosis.^5–7^ Atherosclerosis is a systemic vascular disease representing the aging process and the cumulative adaptive response to cardiovascular risk factors (e.g. hypertension, diabetes).^7–9^ Carotid intima-media thickness (CIMT) is a non-invasive, surrogate marker of subclinical atherosclerosis, associated with cardiovascular risk factors, events and mortality.^8–10^ There is evidence for a positive association between long-term ambient PM and CIMT.^11^^,^^12^ However, the magnitude of associations has been heterogeneous and studies are limited to high-income countries with low or moderate levels of air pollution.^12^^,^^13^ Additionally, the evidence for the association between HAP and CIMT is limited.^14^

The Cardiovascular Health effects of Air pollution in Telangana, India (CHAI) project aims to address the gap by providing evidence on the association between PM and cardiovascular risk (i.e. CIMT) in a population from a LMIC with higher exposure levels than previously reported in the literature.^12^^,^^13^ The primary objective of this study was to quantify the association between ambient annual exposure to PM_2.5_ (particulate matter with an aerodynamic diameter of ≤2.5 µm) and CIMT in India. Our secondary objective was to test whether exposure to biomass cooking fuel, a surrogate of HAP, was associated with CIMT.

## Methods

### Study design and participants

The design of the Andhra Pradesh Children and Parent Study (APCAPS) and CHAI has been described previously.^13^^,^^15^ Briefly, APCAPS is a large prospective, intergenerational cohort that began with the long-term follow-up of the Hyderabad Nutrition Trial (1987–1990). We used data from the third follow-up, which surveyed 6944 participants from 2386 households situated in 28 villages in a peri-urban area south of the city of Hyderabad during 2010–2012. CHAI builds on the APCAPS cohort by extensively characterizing air pollution exposure in the APCAPS area.^13^ We included adults (age ≥18 years), men and non-pregnant women, with non-missing age, gender and CIMT.

APCAPS was approved by the London School of Hygiene & Tropical Medicine (London, UK) and the National Institute of Nutrition (NIN) (Hyderabad, India). CHAI was approved by the Ethics Committees of Parc de Salut MAR (Barcelona, Spain), the Indian Institute of Public Health (Hyderabad, India) and the NIN. Signed consent forms were obtained from all participants.

### Procedures

Data collection was conducted primarily at clinics established within the villages. Data were collected via standardized questionnaires, assessing demographic, socio-economic status [education, occupation, Standard of Living Index (SLI)],^15^ health behaviours (smoking, environmental tobacco smoke, alcohol intake, diet and physical activity),^15^ medical history and household characteristics. Anthropometric measurements, blood pressure and fasting blood samples were collected following standard procedures.^15^ During the first clinic visit in villages, all participants were invited to attend a second clinic visit at the NIN, located in Hyderabad. Participants were offered transport from each village to attend the clinic at NIN. At the NIN, the APCAPS team collected data on cardiovascular outcomes, including CIMT.

### Outcomes

CIMT was measured by a trained physician following the recommended guidelines, using a B-mode ultrasound scanner (Ethiroli Tiny-16a, Surabhi Biomedical Instrumentation, India), at the right common carotid, close to the bulb. CIMT was analysed using semi-automated software which read a 10 mm segment from the near (i.e., the artery wall close to the ultrasound probe) and far walls.^8^ Our primary outcome was mean CIMT, which was calculated as the mean CIMT of available measurements (median of two per individual; all participants had far wall measurements and half also near wall). Our secondary outcome was maximum CIMT, which was the maximum thickness measured for each participant.

### Exposure assessment

We estimated annual ambient concentration of PM_2.5_ at residence using a land-use regression (LUR) model developed for the study area. The measurements and modelling approach have been detailed elsewhere.^13^^,^^16^ Briefly, we measured ambient concentrations of PM_2.5_ (24-h integrated gravimetric PM_2.5_ for 21 days over two seasons) at 23 sites located within 16 of the area villages between 2015 and 2016. Site selection aimed to maximize contrast in predictors of PM; the 12 villages not sampled had similar characteristics to those with measurements.^13^^,^^16^ The PM_2.5_ LUR model included tree coverage, night-time light intensity, longitude and normalized difference in vegetation index,^16^ and explained 58% (mean adjusted *R*^2^) of variation in measured PM_2.5_.

We used self-reported data on cooking fuel as a proxy for HAP. We derived a binary variable for biomass fuel use by grouping participants with primary fuels with relatively high emissions of air pollution (crop residues/dung/wood, 97.7%; kerosene, 2.1%; oil, 0.2%), which we refer to as ‘biomass’ compared with participants using clean fuel (gas, 97.4%; electricity, 2.6%).

Further definitions about HAP, stove ventilation and covariates are shown in Supplementary Table 1, available as Supplementary data at *IJE* online.

### Statistical analysis

Prior to data collection, we estimated 97% power to detect a 1% increase in CIMT for a 1 µg/m^3^ increase in PM_2.5_, assuming *n* = 4000, alpha = 5% and standard deviation (SD) = 3 µg/m^3^ for PM_2.5._^13^

We assessed the effect of PM_2.5_ and biomass fuel on CIMT fitting a ‘within-between’ model.^17^ This model is suitable for scenarios with modest within-group variability in exposure and when the random effect is correlated with the exposure.^17^ The ‘within-between’ specification also has the advantage of accounting for confounding at the village level.^17^^,^^18^ The ‘within-between’ specification was well suited for our data: we observed a correlation between village and ambient PM_2.5_ and a negative association between village-average PM_2.5_ and CIMT (Supplementary Figure 1, available as Supplementary data at *IJE* online), suggesting important between-village confounding. All models were fitted with log transformation of CIMT because of residuals distribution, and included a nested random intercept (households nested within villages).

We selected potential confounders according to a directed acyclic graph based on the literature. Our main analysis was conducted in the whole cohort, followed by gender-stratified analysis due to observed differences in activity patterns, exposure determinants and the distribution of confounders.^19^^,^^20^ We performed sequential adjustment for potential confounders: Model 1, adjusted for age (modelled with a natural spline, degrees of freedom (df) = 3) and gender; Model 2, additionally adjusted for occupation, education, SLI, body mass index, smoking status, environmental tobacco smoke, alcohol, fruits and vegetable consumption, and physical activity; Model 3 (main model), additionally included co-exposure to biomass fuel and stove ventilation. Models for women did not include active smoking because few women reported active smoking (0.3%). The only interaction term defined a priori was between biomass fuel use and stove ventilation.^21^ We applied inverse probability weighting to deal with potential selection bias,^22^ because not all participants were available to travel to Hyderabad for the CIMT measurement. We used multiple imputation for missing covariate data.^22^ Further description of inverse probability weighting and multiple imputation are included in the Supplementary data, available at *IJE* online.

To further explore potential effect modification, we evaluated the effect of PM_2.5_ on our primary outcome according to pre-specified subgroups (e.g. participants ≥40 years old, those with or without diabetes).^11^^,^^18^^,^^23^ We also investigated the interaction between age, gender and biomass use to explore observed differences in CIMT between men and women.

We conducted four sensitivity analyses to assess the robustness of our findings. First, we included in our main model three mediators [blood pressure, impaired fasting glucose and non-high-density lipoprotein (HDL) cholesterol] to evaluate if the association was attenuated as expected. Second, we fitted our main model on multiple imputed data without selection bias correction, and, finally, on complete-case data with and without selection bias correction.

We followed STrengthening the Reporting of OBservational studies in Epidemiology (STROBE) guidelines. Analyses were conducted with R-3.4.2, with the packages *tidyverse*, *mice*, *miceadds*, *lme4*, *metafor*, *ggplot2* and *forestplot*.

## Results

Among those eligible for inclusion (*n* = 6229), 3445 (55%) attended the second clinic visit (Supplementary Figure 2, available as Supplementary data at *IJE* online). Participants who attended the NIN had a higher prevalence of known risk factors for cardiovascular diseases (Supplementary Table 2, available as Supplementary data at *IJE* online). Of the 3445 participants, we excluded 51 (1.5%) because of missing residential address and 116 (3.4%) because of missing CIMT measurements, resulting in 3278 participants included in the analysis.

### Participant characteristics

Table 1 describes the cohort. Mean age was 38 (SD = 14) years and 50% of participants were ≥40 years. Most women had no formal education (70%). One-third of participants were overweight. The prevalence of hypertension and impaired fasting glucose/diabetes was ∼25% and we observed a high prevalence of dyslipidaemias, particularly low HDL cholesterol (59% overall, 71% for women). Among participants ≥40 years, one-third had metabolic syndrome according to the harmonized metabolic syndrome definition.^24^ Regarding health behaviours, 556 (33%) men were current smokers, whereas 658 (52%) of women were exposed to environmental tobacco smoke.

**Table 1.** Participant characteristics overall and stratified by gender

Variable	Category	All (*n* = 3278)	Men (*n* = 1693)	Women (*n* = 1585)
Age (years)	Mean	38 (14)	37 (16)	38 (12)
Age (categories)	18.0–29.9	1349 (41.2%)	839 (49.6%)	510 (32.2%)
	30.0–39.9	307 (9.4%)	106 (6.3%)	201 (12.7%)
	40.0–49.9	878 (26.8%)	243 (14.4%)	635 (40.1%)
	50.0–59.9	563 (17.2%)	349 (20.6%)	214 (13.5%)
	60.0–	181 (5.5%)	156 (9.2%)	25 (1.6%)
Gender	Women	1585 (48.4%)	–	1585 (100%)
Education^a^	No formal education	1781 (54.3%)	672 (39.7%)	1109 (70.0%)
	Primary (1–4 years)	413 (12.6%)	268 (15.8%)	145 (9.1%)
	Secondary (5–12 years)	874 (26.7%)	599 (35.4%)	275 (17.4%)
	Beyond secondary (>12 years)	209 (6.4%)	153 (9.0%)	56 (3.5%)
Occupation^a^	Unemployed	811 (24.7%)	332 (19.6%)	479 (30.2%)
	Unskilled manual	1668 (50.9%)	747 (44.1%)	921 (58.1%)
	Skilled manual	661 (20.2%)	503 (29.7%)	158 (10.0%)
	Non-manual	137 (4.2%)	110 (6.5%)	27 (1.7%)
Standard of living index^a^	Low (0–14)	163 (5.0%)	72 (4.3%)	91 (5.8%)
	Medium (15–24)	999 (30.9%)	490 (29.4%)	509 (32.6%)
	High (25–67)	2066 (64.0%)	1105 (66.3%)	961 (61.6%)
Comorbidities				
Body mass index (kg/m^2^)^a^	Underweight (<18.5)	994 (30.4%)	557 (32.9%)	437 (27.6%)
	Normal weight (18.5–22.9)	1434 (43.8%)	760 (44.9%)	674 (42.6%)
	Overweight (23.0–24.9)	405 (12.4%)	184 (10.9%)	221 (14.0%)
	Obese (25.0–)	442 (13.5%)	190 (11.2%)	252 (15.9%)
Central obesity	Waist circumference ≥80 cm for women and ≥90 cm for men	427 (13.0%)	132 (7.8%)	295 (18.6%)
Hypertension	SBP ≥140 mmHG or DBP ≥90 mmHg or anti-hypertensive intake	705 (21.5%)	427 (25.2%)	278 (17.6%)
Glucose intolerance	Impaired fasting glucose	731 (22.3%)	390 (23.0%)	341 (21.5%)
	Diabetes	169 (5.2%)	100 (5.9%)	69 (4.4%)
Lipid profile^a^	Total cholesterol ≥200 mg/dL	536 (16.7%)	271 (16.1%)	265 (17.2%)
	HDL cholesterol <50 mg/dL for women and <40 mg/dL for men	1901 (59.1%)	810 (48.2%)	1091 (70.9%)
	Non-HDL cholesterol ≥130 mg/dL	1174 (36.5%)	603 (35.9%)	571 (37.1%)
	Triglycerides ≥150 mg/dL	759 (23.7%)	472 (28.3%)	287 (18.7%)
Metabolic syndrome	≥3 criteria	616 (18.8%)	302 (17.8%)	314 (19.8%)
Health behaviours				
Smoking status^a^	Never	2717 (82.9%)	1136 (67.1%)	1581 (99.7%)
	Former	32 (1.0%)	32 (1.9%)	–
	Current	528 (16.1%)	524 (31.0%)	4 (0.3%)
	Age started smoking (years)	25 (10)	25 (10)	–
Environmental tobacco smoke	Yes	1093 (33.3%)	435 (25.7%)	658 (41.5%)
Alcohol use^a^	Most days	992 (30.3%)	720 (42.6%)	272 (17.2%)
Diet	Percentage of energy from carbohydrates	67.9% (10)	66.8% (11)	69.1% (8)
	Percentage of energy from fat	17.0% (5)	16.1% (5)	17.9% (6)
	Percentage of energy from saturated fat	4.8% (2)	4.5% (2)	5.0% (2)
	Percentage of energy from protein	9.2% (2)	8.9% (2)	9.4% (1)
Physical activity (METs)^a^	Sedentary or light activity (<1.70)	2047 (64.7%)	1161 (70.8%)	886 (58.1%)
	Active or moderately active (1.70–1.99)	926 (29.3%)	395 (24.1%)	531 (34.8%)
	Vigorously active (>2)	192 (6.1%)	84 (5.1%)	108 (7.1%)
Fuel use				
Main source of cooking fuel^a^	Biomass	1937 (60.1%)	957 (57.5%)	980 (62.9%)
Stove ventilation	Not vented to the outside	841 (25.7%)	443 (26.2%)	398 (25.1%)
Main source of lighting fuel^a^	Biomass	43 (1.3%)	17 (1.0%)	26 (1.6%)

a Missing values were 1 (<0.1%) for occupation, education, smoking status, alcohol use and lighting fuel use, 2 (<0.1%) for hypertension, 3 (<0.1%) for body mass index, 4 (0.1%) for abdominal obesity, 50 (1.5%) for standard living index, 54 (1.6%) for main source of cooking fuel, 59 (1.8%) for cholesterol, 76 (2.3%) for triglycerides, 113 (3.4%) for physical activity. Data are mean (SD) or *n* (%).

DBP, diastolic blood pressure; HDL, high-density lipoprotein; METs, metabolic equivalents; SBP, systolic blood pressure.

### Distribution of air pollution and carotid intima-media thickness

**Figure 1.**
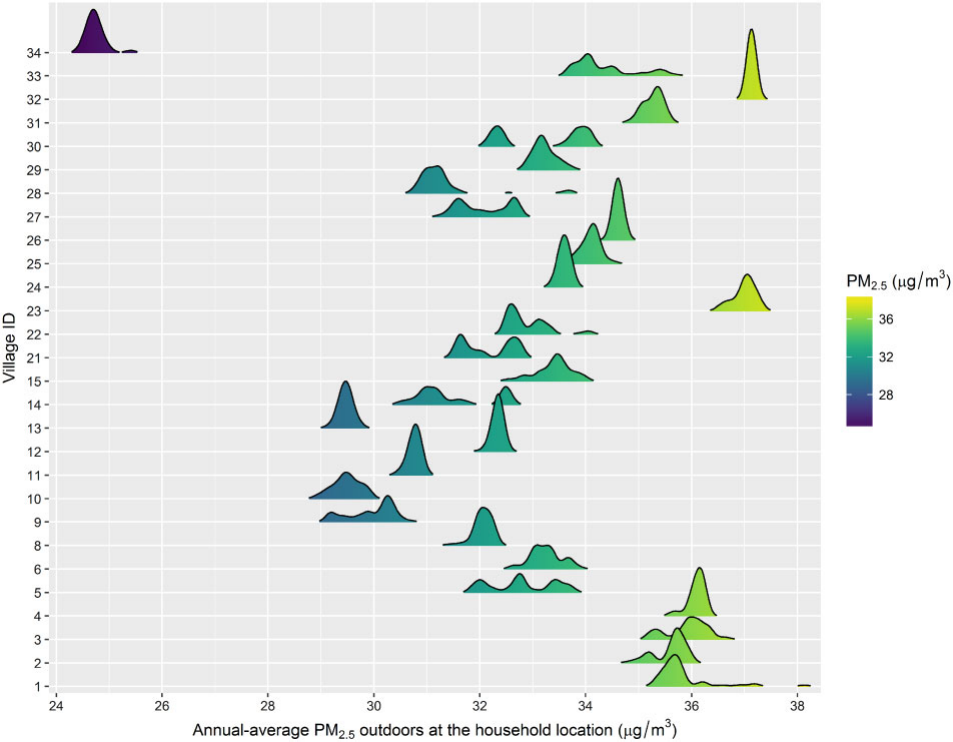
Predicted concentrations of annual average PM_2.5_ (µg/m^3^) outdoors at the household location stratified by village. The concentrations were derived from a validated land-use regression model. The distribution of PM_2.5_ for each village is presented as a density plot, drawn using a kernel density estimate. PM_2.5,_ particulate matter with an aerodynamic diameter of ≤2.5 µm.

Figure 1 shows the village distribution of annual PM_2.5_. Mean annual PM_2.5_ was 32.7 µg/m^3^ (interquartile range = 3.2), varying from 24.4 to 38.2 µg/m^3^ (range 13.8). Biomass fuel was reported as the main cooking fuel for 60% of participants and 26% of participants did not have the stove vented to the outside (Table 1).

The mean CIMT was 0.829 mm (SD = 0.252) and maximum CIMT 0.865 mm (SD = 0.273). Women had higher unadjusted CIMT compared with men [0.063 mm mean CIMT, 95% confidence interval (CI), 0.046–0.080; 0.066 mm maximum CIMT, 95% CI, 0.048–0.085] (Table 2). Participants using biomass cooking fuel had 0.048 mm higher unadjusted mean CIMT (95% CI, 0.030–0.065) and 0.051 mm higher maximum CIMT (95% CI, 0.032–0.070) compared with participants using gas/electricity.

**Table 2.** Association of within-village variation in PM_2.5_ and biomass fuel use with mean and maximum carotid intima-media thickness in all participants and stratified by gender. Analysis conducted in ten multiple imputed datasets, using a linear mixed model accounting for within-between effects, with correction for selection bias through inverse probability weighting

Model	Exposure	All (*n* = 3278)	Men (*n* = 1693)	Women (*n* = 1585)
Outcome: mean CIMT (mm)		0.829 (0.25)	0.799 (0.26)	0.862 (0.24)
		Percent difference in CIMT (95% CI)	Percent difference in CIMT (95% CI)	Percent difference in CIMT (95% CI)
Ambient air pollution				
Model 1 (basic adjustment)^a^	PM 2.5 (1 µg/m^3^)	1.88 (−0.26, 4.01)	3.05 (0.27, 5.84)	0.54 (−2.41, 3.49)
Model 2 (full adjustment)^b^	PM 2.5 (1 µg/m^3^)	1.80 (−0.31, 3.91)	3.02 (0.28, 5.77)	0.52 (−2.41, 3.44)
Model 3 (Model 2 + HAP)^c^	PM 2.5 (1 µg/m^3^)	1.79 (−0.31, 3.90)	2.98 (0.23, 5.72)	0.51 (−2.40, 3.43)
Household air pollution				
Model 3 (Model 2 + HAP)^c^	Biomass	1.60 (−0.46, 3.65)	1.77 (−0.89, 4.44)	
	Biomass (Vented)			−0.20 (−3.35, 2.95)
	Biomass (Not vented)			6.14 (1.40, 10.89)
Outcome: maximum CIMT (mm)		0.865 (0.27)	0.833 (0.28)	0.899 (0.26)
Ambient air pollution				
Model 1 (basic adjustment)^a^	PM 2.5 (1 µg/m^3^)	2.07 (−0.17, 4.30)	3.67 (0.75, 6.60)	0.28 (−2.83, 3.39)
Model 2 (full adjustment)^b^	PM 2.5 (1 µg/m^3^)	1.99 (−0.23, 4.20)	3.59 (0.69, 6.48)	0.26 (−2.83, 3.35)
Model 3 (Model 2 + HAP)^c^	PM 2.5 (1 µg/m^3^)	1.98 (−0.24, 4.20)	3.54 (0.65, 6.44)	0.24 (−2.85, 3.32)
Household air pollution				
Model 3 (Model 2 + HAP)^c^	Biomass	1.62 (−0.57, 3.81)	1.73 (−1.08, 4.54)	
	Biomass (vented)			−0.18 (−3.52, 3.16)
	Biomass (not vented)			5.44 (0.42, 10.47)

a Model 1 was adjusted by age (modelled with natural spline, df = 3) and sex.

b Model 2: Model 1+ occupation, education, standard of living index, body mass index, fruits and vegetables consumption, smoking status and environmental tobacco smoke, alcohol consumption and physical activity. Models for women did not include active smoking.

c Model 3: Model 2+ biomass fuel use and whether stove was vented to the outside. The models for women have an interaction term between biomass fuel use and whether stove was vented to the outside.

CI, Confidence interval; CIMT, carotid intima-media thickness; HAP, household air pollution; PM_2.5_ = particulate matter with an aerodynamic diameter of ≤2.5 µm.

### Association between air pollution and carotid intima-media thickness

We observed a positive association with limited precision between within-village variation in annual PM_2.5_ and CIMT in the whole population (1.79%, 95% CI, −0.31, 3.90, per 1  µg/m^3^ of PM_2.5_) after adjustment for potential confounders (Table 2). This association was primarily driven by men (2.98, 95% CI, 0.23–5.72, per 1  µg/m^3^ of PM_2.5_). There was no evidence that the effect of annual PM_2.5_ was non-linear across the exposure range in the study population. There was a positive, but imprecise, association of biomass compared with clean fuel (1.60%, 95% CI, −0.46–3.65, Model 3) in the whole population and a larger association among women with an unvented stove (6.14%, 95% CI, 1.40–10.89) (Table 2).

### Effect modification

**Figure 2.**
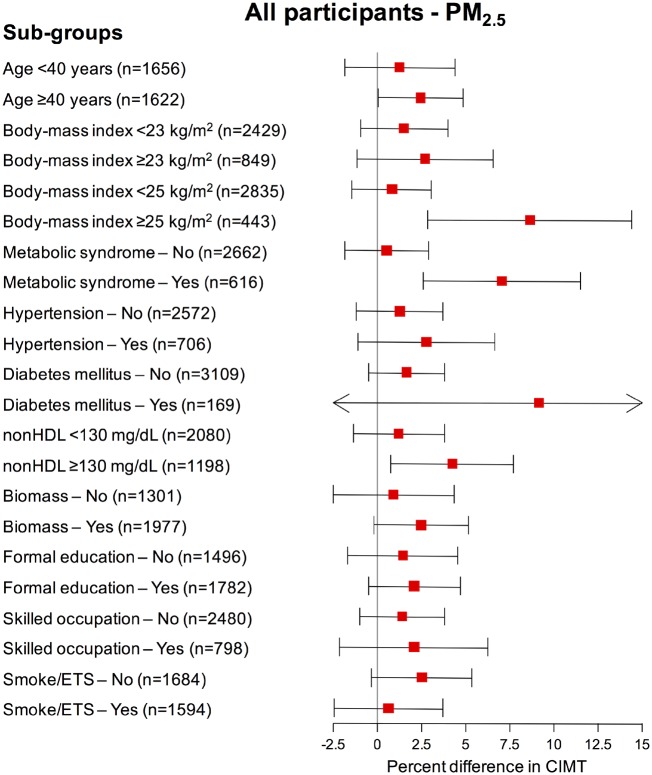
Association of within-village variation in PM_2.5_ with mean carotid intima-media thickness stratified by participant characteristics. The adjusted percent difference in carotid intima-media thickness was estimated per 1 µg/m^3^ of PM_2.5_. The point-estimates are represented by boxes and their 95% confidence intervals as horizontal lines. The arrows represent the direction of the confidence interval estimation, truncated at the horizontal axes limit. Estimations were retrieved from our main model (Model 3). Model 3 was adjusted by age (natural spline, df = 3), gender, occupation, education, standard of living index, body mass index, fruits and vegetables consumption, smoking status and environmental tobacco smoke, alcohol consumption, biomass fuel use and whether stove was vented to the outside. CIMT, carotid intima-media thickness; ETS, environmental tobacco smoking; nonHDL, non-high-density lipoprotein cholesterol; PM_2.5,_ particulate matter with an aerodynamic diameter of ≤2.5 µm.

Associations of annual PM_2.5_ with mean CIMT (Model 3) according to subgroups are presented in Figure 2. The point-estimates for the association between annual PM_2.5_ and CIMT were larger for those aged ≥40 years (2.47%, 95% CI, 0.08, 4.86, per 1  µg/m^3^ of PM_2.5_) and for participants with high cardiometabolic risk (e.g. obesity, metabolic syndrome and dyslipidaemia).

**Figure 3.**
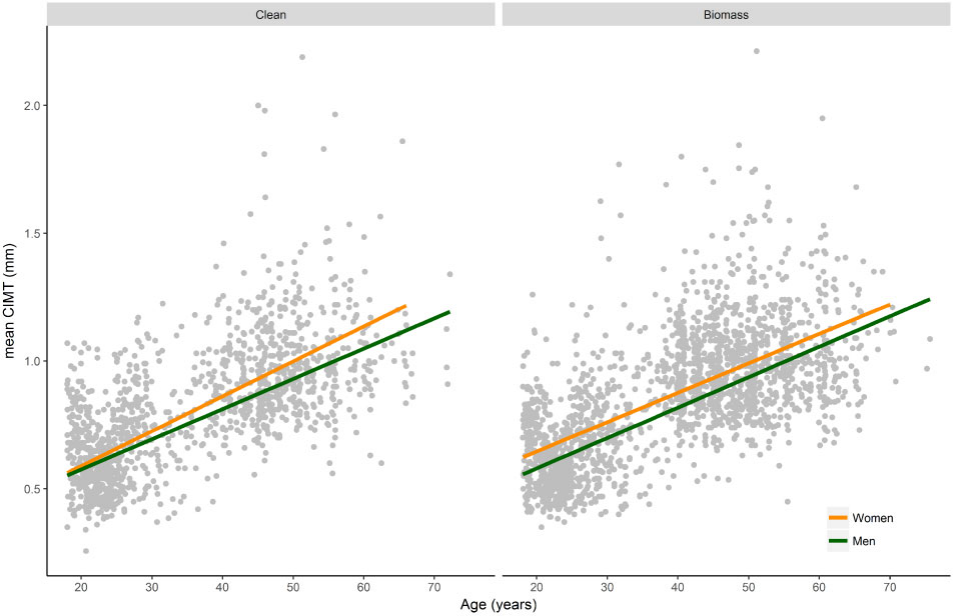
Predicted mean carotid intima-media thickness taking into account the interaction among age, gender and cooking fuel. Predicted mean carotid intima-media thicknesses (geometric means) were retrieved from a model including an interaction term for age, gender and biomass fuel, and adjusting for occupation, education, standard of living index, body mass index, fruits and vegetables consumption, smoking status and environmental tobacco smoke and alcohol consumption. Lines represent the predicted mean carotid intima-media thickness from the model; dots are the original mean carotid intima-media thickness values. CIMT, carotid intima-media thickness.

When exploring the interaction between age, gender and HAP, we observed that men and women had comparable mean CIMT among young participants exposed to clean fuel. However, women had higher mean CIMT compared with men for all ages among those exposed to biomass [*P*_interaction (age x gender x biomass)_ = 0.005] (Figure 3).

### Sensitivity analysis

As expected, the point-estimate for annual PM_2.5_ decreased after adding mediators to the model (Supplementary Table 3, available as Supplementary data at *IJE* online), but mediators did not fully explain the observed relationship. Regarding other sensitivity analyses, results were comparable to the main analysis, with minor changes Supplementary Tables 3, 4 and 5, available as Supplementary data at *IJE* online).

## Discussion

We observed a positive association between annual ambient PM_2.5_ and CIMT, with larger estimates for men, participants ≥40 years, or those with cardiometabolic risk factors. Additionally, biomass cooking fuel was associated with higher CIMT, with stronger evidence for women cooking with unvented stoves. Finally, women had higher CIMT than men, which might be associated with air pollution exposure from biomass cooking fuel.

We observed a relatively large point-estimate for the association between annual ambient PM_2.5_ and CIMT compared with previous studies in high-income countries.^12^^,^^25^ Indeed, the point-estimate in percent-change of CIMT after standardizing by 5 µg/m^3^ increase of PM_2.5_ was 1.66% (95% CI, 0.86–2.46%) in a meta-analysis with eight studies,^12^ whereas our estimate was 8.98%. One potential explanation for this finding is the high cardiometabolic risk profile observed in the study population, particularly considering the relatively young age. The population characteristics and overall estimate are comparable with findings in high-risk subgroups from previous studies, such as in the seminal study conducted in the USA for older women and those taking lipid-lowering therapy,^11^ and recently reported in Switzerland for participants with diabetes, hypertension and high cardiovascular risk.^23^ Other epidemiological and experimental studies support the rationale for a greater harmful effect of ambient air pollution on cardiovascular health when the underlying risk is high, such as dyslipidaemia.^5^^,^^26^ Compared with other populations, a relatively large proportion of participants in our study with hypertension or diabetes had not received a previous diagnosis (70% for hypertension, 46% for diabetes) for the condition, suggesting uncontrolled cardiovascular risk factors may increase susceptibility to the adverse effects of air pollution.

Another potential explanation for the large magnitude of the point-estimate for the association between ambient PM_2.5_ and CIMT may be the relatively high levels of exposure to annual ambient PM_2.5_ in this population (mean 32.7 µg/m^3^) compared with previous studies: mean PM_2.5_ varied from 7.6 to 20.7 µg/m^3^ in a meta-analysis with eight studies;^12^ and, mean PM_2.5_ was 27.3 µg/m^3^ in a study in Taiwan.^27^ Further studies are needed in populations with high average levels of PM_2.5_ to better determine whether the association between ambient PM_2.5_ and CIMT is non-linear over a wide exposure range. The sources, chemical composition and toxicity of ambient PM_2.5_ in our study area likely differ compared with those in populations in urban areas of high-income countries,^12^^,^^13^^,^^28^^,^^29^ which might also explain differences in the magnitude of point-estimates across populations.

About three billion individuals are exposed to HAP.^30^ Nevertheless, the relationship between HAP and cardiovascular diseases is not well characterized and the attributable burden is uncertain.^30^ In the APCAPS population, 60% of participants used biomass as the main cooking fuel, a source of exposure to PM and other toxic pollutants. We observed a positive association between biomass fuel use and CIMT, especially among women with an unvented stove. Women in our population reported cooking for a median of 2 h per day, and we previously showed cooking with a biomass stove increased personal exposure to PM_2.5._^31^ Moreover, lack of ventilation has been associated with higher exposure to air pollutants and risk of cardiovascular events.^21^^,^^32^

We also observed that women had consistently higher CIMT than men for all ages, contrasting with other populations.^10^^,^^12^ We explored whether this difference might be due to exposure to HAP. Indeed, we observed that for participants <30 years old, the gender difference was minimal among those not exposed to biomass; however, women had higher CIMT than men at young ages in those exposed to biomass. The household energy transition from traditional biomass burning cookstoves to clean cooking fuels (e.g. liquid petroleum gas) has been recent in this area.^33^ Therefore, older participants, particularly women, would have been exposed to HAP for many years in the past, likely contributing to the higher CIMT in women compared with men at older ages.

Our findings reinforce the need for studies from LMICs, which may differ substantially from high income countries in terms of population characteristics as well as levels and sources of particulate air pollution. To illustrate the population impact of ambient annual PM_2.5_ in the studied population, its point-estimate corresponds to approximately one-third the effect observed for active tobacco smoking. This increase in risk is important, particularly given that the entire population is exposed to ambient air pollution. Two recent systematic reviews highlighted the lack of evidence for long-term exposure to ambient PM and HAP and their effects on cardiovascular diseases in LMICs.^14^^,^^34^ Our study evaluates the highest average levels of ambient PM_2.5_ in relation to CIMT in the literature to date.^12^^,^^25^ Regarding HAP, to the best of our knowledge, we report the largest study on HAP and CIMT,^14^^,^^30^^,^^35^ shedding light on possible explanations for a recent prospective cohort study in China showing strong evidence for solid fuel use and cardiovascular mortality in 271 217 adults.^32^

Our study analysed a population representative of the source population within these 28 villages,^36^ and applied a locally developed LUR model to estimate exposure to annual ambient air pollution outdoors at the residential address in a peri-urban area in India.^16^ Nonetheless, some limitations should be considered when interpreting our results. First, the current analysis is cross-sectional, limiting our ability to investigate the role of air pollution in CIMT progression. Second, our estimate of exposure (annual ambient PM_2.5_) does not represent the cumulative life-time exposure to ambient PM_2.5_ and might not be representative of long-term average personal exposure to PM_2.5_. Additionally, we did not have participants’ lifetime residential history, although based on available data we estimate that migration within and away from the APCAPS villages is low (∼2%). Because the exposure measurement was done in 2015–2016 and the outcome measurement in 2010–2012, we used the geographical predictors in the LUR model from 2012/2013 and assumed that the spatial pattern of ambient air pollution sources remained constant between 2010 and 2015.^16^^,^^36^ Third, CIMT is challenging to measure.^8^ We used a standardized procedure to measure CIMT, thereby improving the internal validity of our findings; however, direct comparisons of our measurements with CIMT values reported in the literature are limited by differences in measurement protocols. Some exposure measurement error is likely, both from the LUR and self-reported HAP, which might have biased our estimates towards the null. Although we adjusted for several potential confounders, we cannot rule out residual confounding. Part of this concern is minimized because the ‘within-between’ model accounts for unmeasured factors at the village level, and differences in estimates from models with basic and full confounder adjustment were generally modest. Additionally, we adjusted for mediators in sensitivity analyses and our estimates were partially attenuated, consistent with the hypothesized biological pathways. Finally, we observed a lower level of annual ambient PM_2.5_ than occurs in cities in Northern India (e.g. New Delhi), and lower prevalence of central obesity and metabolic syndrome than expected for urban populations in India.^37^ Therefore, generalizability of our estimates to urban India may be somewhat limited, and further studies are needed to better represent other populations in LMICs.^28^

## Conclusions

Our results provide evidence that exposure to annual ambient PM_2.5_ is associated with CIMT among men in a population with a high prevalence of cardiometabolic risk factors. Moreover, the use of biomass fuel was associated with CIMT among women cooking with unvented stoves. Our results contribute new evidence of the effect of annual PM_2.5_ on CIMT at relatively high exposures.

## Funding

This work was supported by the European Research Council (Grant Agreement 336167 for the CHAI Project) and the Wellcome Trust (Grant: 084674/Z for the third follow-up of the APCAPS). C.T. was funded through a Ramón y Cajal fellowship (RYC-2015–17402) awarded by the Spanish Ministry of Economy and Competitiveness.

## Supplementary Material

dyz208_Supplementary_Data
