# Supplementary material for: Association between ambient and household air pollution with carotid intima-media thickness in peri-urban South India: CHAI-Project
Source: Int J Epidemiol. 2019 Oct 11;49(1):69–79. doi: 10.1093/ije/dyz208 (PMC7124504; doi:10.1093/ije/dyz208)
Supplement: dyz208_Supplementary_Data [file dyz208_supplementary_data.pdf]

Supplemental content

Association between ambient and household air pollution with carotid intima-media thickness in peri-urban South India: CHAI-Project

eTable 1. **Definitions of household air pollution exposure and covariates**

Variable	Definition	Questionnaire
**Household air pollution exposure**		
Main source of cooking fuel	We categorized those using biomass (c, d, and e) vs no biomass (a and b)	What is the main source of cooking fuel? Options: a) Electricity, b) Gas, c) Kerosene, d) Oil, e) Other.<br><br>Note: For multiple sources, field team recorded the predominant one.<br>Other includes wood, crop residues or dung.
Stove ventilation	We kept the same categories as the questionnaire Those with stove located outside were considered vented.	Was your stove or fire vented to the outside? Options: a) Yes, b) No.
Main source of lighting fuel	We categorized those using biomass (c, d, and e) vs no biomass (a and b)	What is the main source of lighting for your household? Options: a) Electricity, b) Gas, c) Kerosene, d) Oil, e) Other.<br><br>Note: For multiple sources, field team recorded the predominant one. Other includes wood, crop residues or dung.
**Covariates**		
Education	We categorized in No formal education (a and b), Primary (1-4 years) (c), Secondary (5-12 years) (d) and Beyond secondary (>12 years)(e and f)	Highest education level attained. Options: a) Illiterate, b) Literate, no formal education, c) Up to primary school (Class IV), d) Secondary School (ITI Course, Class/XII, Intermediate), e) Graduate (BA, B.Sc., B.Com, Diploma), f) Professional degree/postgraduate(M.A., MSc, MBBS, MSW, Btech, PhD)
Occupation	We categorized in Unemployed (a, b, c and d), Unskilled manual (e), Skilled manual (f and g) and Non-manual (h, i and j)	Primary occupation: a) At home doing housework, b) Unemployed, not seeking work; retired/ disabled, c) Unemployed, seeking work, d) Student/ training, e) Unskilled manual, f) Semi-skilled manual, g) Skilled manual, h) Skilled non-manual, i) Semi-Professional, j) Professional
Standard Living Index	We used the this standard index for socioeconomic status assessment validated for use in India.[1,2]	Household-level, asset-based questionnaire
Abdominal obesity	Waist circumference ≥ 80 cm for women and ≥90 cm for men.[3]	
Hypertension	Systolic blood pressure ≥140 mmHG or diastolic blood pressure ≥90mmHg or anti-hypertensive intake	Are you on regular medication for your high blood pressure? Options: a) Yes, b) No.
Impaired fasting glucose	Fasting plasma glucose level ≥ 100 mg/dL	
Diabetes	Fasting plasma glucose level ≥ 126 mg/dL or diabetes diagnosed	Have you been diagnosed with diabetes?<br>Options: a) Yes, b) No.
Metabolic syndrome	At least 3 of the following criteria:[3] a) abdominal obesity, b) systolic blood pressure ≥130 mmHG or diastolic blood pressure ≥85mmHg or anti-hypertensive intake, c) impaired fasting glucose, d) HDL cholesterol <50 mg/dL for women and <40 mg/dL for men, e) Triglycerides ≥150 md/dL	
Tobacco smoke	We kept the same categories as from the questionnaire.	Have you ever used tobacco on a regular basis (at least weekly)? Options: a) Never, b) Former (stopped over 6 months ago), c) Current (anytime in the last 6 months).
Environmental tobacco smoking	We categorized participants in those exposed (a) or not (b).	Is there someone in your household who smokes tobacco at home? Options: a) Yes, b) No.
Alcohol use	We categorized participants in those who drank most days (a and b) and Rarely/Never (c, d and e)	Would you describe your present alcohol intake as? (Included locally made spirits / Branded made spirits / Beer / Wine) Options: a) Daily/Most days, b) Weekends, c) 1-2 times/month, d) Special occasions, e) Never.
Diet	The percentage of energy from each food category, as well as fruits and vegetable amount, were derived from a validated questionnaire.[4]	Semi-quantitative food frequency questionnaire (FFQ)
Physical activity	We used the metabolic equivalent unit values (MET) derived from the validated questionnaire.[5]	Interviewer-administered quantitative physical activity questionnaire

Further description of statistical analysis

Of the 6944 participants enrolled in the third follow-up of Andhra Pradesh Children and Parent Study (APCAPS) cohort (first clinic visit established within the villages), we included adults (age ≥ 18 years), men and non-pregnant women, with available CIMT measurement. Among those eligible for inclusion (n=6229), 3445 (50%) attended the second clinic visit at the National Institute of Nutrition (NIN) located in Hyderabad , for the cardiovascular risk profile measures (eFigure 1, Study flowchart). There were differences between those who attended the clinic visit at NIN and those who did not. Participants who attended the NIN clinic visit had a higher prevalence of known risk factors for cardiovascular diseases (eTable 2). To adjust for potential selection bias, we used inverse probability weighting (IPW) technique to account for the population representativeness.[6,7] The IPW were applied in two steps a) deriving the probability of attending the NIN for each individual and b) estimating the weight for each participant (1/probability of attending), which was subsequently used in the linear mixed model. Therefore, each participant contribution to the model is weighted, to achieve estimates that aim to be representative of the source population (i.e., participants with a profile that are more likely to attend receive lower weights compared with participants with a profile not attending). Thus, the final model was fit in a pseudopopulation, generated with the derived weights.[6,7]

We followed the published recommendations for the IPW generation.[6,8] The model to derive the attending probability to the NIN was build using a logistic regression model, where the outcome was binary Yes/No (Yes - participants who attended NIN, NO – participants who did not attend NIN). We used as covariates variables related to the fact of attending or not, but also related to our main outcome (CIMT), as recommended by the literature.[6,8] The covariates were all those included in the full adjustment model (model 3), with education and occupation as originally collected (education = 6 levels; occupation = 10 levels) and the auxiliary variables systolic blood pressure, diastolic blood pressure, impaired fasting glucose/diabetes, abdominal circumference, and metabolic syndrome criteria. The model also included an interaction term between village-ID, age, and gender, to account for the underlying potential process of not attending the NIN, which we hypothesized to be closely related to each village characteristics (e.g., distance to the NIN), and the participant age and gender accounted within villages. Sensitivity analysis using different auxiliary variables (e.g. distance to the NIN instead of village-ID) showed similar results (data not shown).

We assessed the IPW building process and met all suggested requirements: a) We observed a high degree of overlap between weights among participants who attended or not the clinic, b) We did not observe problems with large weights,[6,8] and c) we used the recommended additional tests to evaluate the model (Hosmer-Lemeshow, p=0.885; Hinkley´s method, p=0.791).[6,8]

We followed the published recommendations for the multiple imputation (MI) followed the IPW generation.[6,8,9] We conducted multiple imputation for covariates using multivariate chained equation methods using the *mice* package in R.[9,10] We investigated the missingness pattern and assumed a Missing at Random (MAR) mechanism. We used all covariates used in the full adjustment model (model 3), the outcome, and auxiliary variables. We generated 10 imputed datasets, with 50 iterations, and the estimates were pooled following the Rubin´s rule. We checked the variables distribution and convergence. To allow for the hierarchical structure of the data, we used the predictive mean matching method for all covariates and entered the village-ID as dummy variables.[11,12]

All analyses were conducted with R-3.4.2,[13] with the packages *tidyverse*,[14] *mice*,[10] *miceadds*,[15] *lme4*,[16,17] *metafor*,[18] *ggplot2*,[19] and *forestplot*.[20]

eFigure 1. **Association between village-average $PM_{2.5}$ and CIMT**

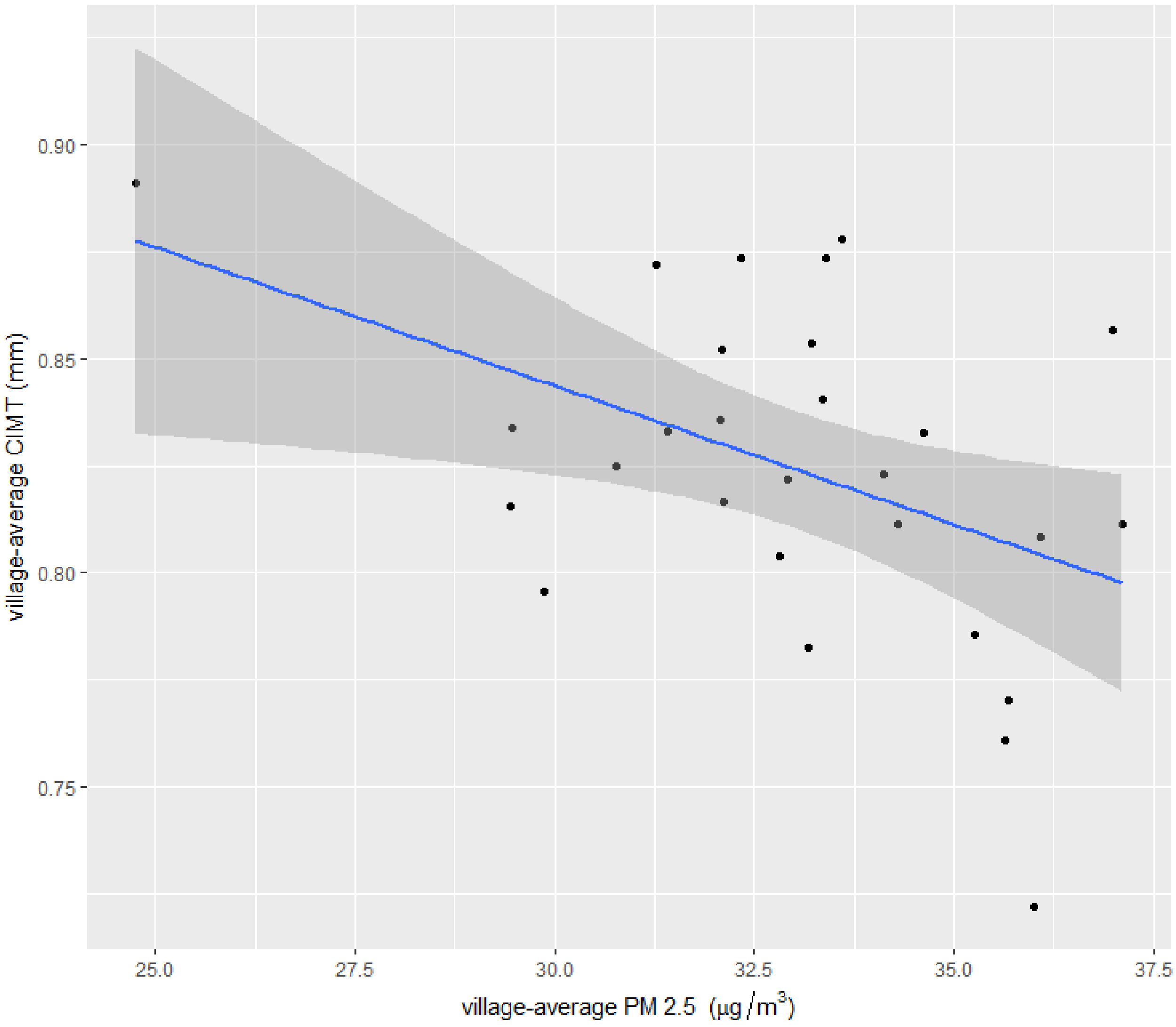

The blue line is the linear regression between village-average PM2.5 and village-average CIMT. The gray zone is the 95% CI. CIMT = carotid intima-media thickness; PM2.5 = particulate matter with an aerodynamic diameter of 2.5 micrometers or less.

eFigure 2. **Study flowchart**

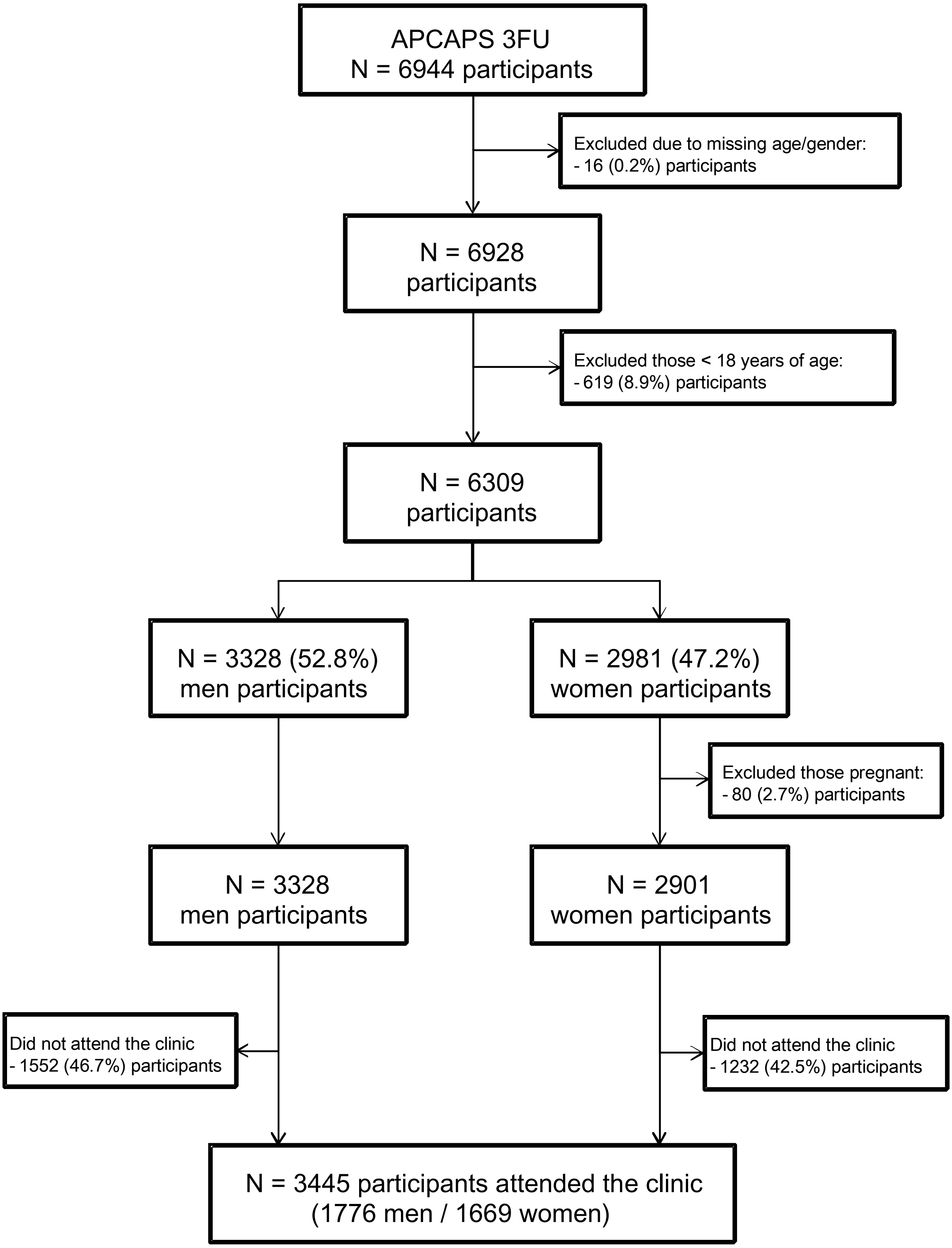

eTable 2. **Participant characteristics according to those who did or did not attend the second clinic visit for carotid intima-media thickness measurements**

**Variable**	**Category**	**All (n = 6229)**	**Did not attend NIN (n=2784)**	**Attended NIN (n=3445)**	**P value**
**Age** (years)	Mean (SD)	36 (14)	34 (14)	38 (14)	<0.001
**Age** (categories)	18.0-29.9	2951 (47.4%)	1538 (55.2%)	1413 (41.0%)	<0.001
	30.0-39.9	581 (9.3%)	260 (9.3%)	321 (9.3%)	
	40.0-49.9	1419 (22.8%)	492 (17.7%)	927 (26.9%)	
	50.0-59.9	952 (15.3%)	367 (13.2%)	585 (17.0%)	
	60.0 -	326 (5.2%)	127 (4.6%)	199 (5.8%)	
**Gender**	Women	2901 (46.6%)	1232 (44.3%)	1669 (48.5%)	0.001
**Education***	No formal education	3047 (48.9%)	1185 (42.6%)	1862 (54.1%)	<0.001
	Primary (1-4 years)	818 (13.1%)	377 (13.5%)	441 (12.8%)	
	Secondary (5-12 years)	1864 (29.9%)	954 (34.3%)	910 (26.4%)	
	Beyond secondary (>12 years)	498 (8.0%)	267 (9.6%)	231 (6.7%)	
**Occupation***	Unemployed	1645 (26.4%)	782 (28.1%)	863 (25.1%)	<0.001
	Unskilled manual	2812 (45.2%)	1069 (38.4%)	1743 (50.6%)	
	Skilled manual	1409 (22.6%)	718 (25.8%)	691 (20.1%)	
	Non-manual	361 (5.8%)	214 (7.7%)	147 (4.3%)	
**Standard living index** (points)*	Low (0-14)	259 (4.3%)	92 (3.5%)	167 (4.9%)	0.002
	Medium (15-24)	1830 (30.3%)	774 (29.1%)	1056 (31.2%)	
	High (25-67)	3952 (65.4%)	1790 (67.4%)	2162 (63.9%)	
**Comorbidities**					
Body-mass index (kg/m^2)*	Underweight (<18.5)	1836 (29.5%)	795 (28.7%)	1041 (30.2%)	0.558
	Normal weight (18.5-22.9)	2740 (44.1%)	1240 (44.7%)	1500 (43.6%)	
	Overweight (23.0-24.9)	760 (12.2%)	338 (12.2%)	422 (12.3%)	
	Obese (25.0 -)	880 (14.2%)	401 (14.5%)	479 (13.9%)	
Abdominal obesity*	Waist circumference ≥ 80 cm for women and ≥90 cm for men	771 (12.4%)	312 (11.3%)	459 (13.3%)	0.015
Hypertension*	SBP ≥140 mmHG or DBP ≥90 mmHg or anti-hypertensive intake	1280 (20.6%)	526 (18.9%)	754 (21.9%)	0.004
Diabetes	Impaired fasting glucose	1388 (22.3%)	591 (21.2%)	797 (23.1%)	0.077
	Diabetes	323 (5.2%)	138 (5.0%)	185 (5.4%)	0.500
Lipid profile*	Total cholesterol ≥200 mg/dL	1032 (17.1%)	458 (17.2%)	574 (17.0%)	0.860
	HDL cholesterol <50 mg/dL for women and <40 mg/dL for men	3502 (57.9%)	1501 (56.3%)	2001 (59.1%)	0.028
	Non-HDL cholesterol ≥130 mg/dL	2278 (37.7%)	1030 (38.6%)	1248 (36.9%)	0.173
	Triglycerides ≥150 md/dL	1428 (23.7%)	621 (23.4%)	807 (24.0%)	0.629
Metabolic syndrome	≥ 3 criteria	1146 (18.4%)	477 (17.1%)	669 (19.4%)	0.022
**Health behaviors**					
Smoking status*	Never	5229 (84.0%)	2382 (85.6%)	2847 (82.7%)	0.003
	Former	49 (0.8%)	15 (0.5%)	34 (1.0%)	
	Current	948 (15.2%)	385 (13.8%)	563 (16.3)	
	Age started smoking (years)	24 (10)	24 (9)	24 (10)	0.797
	Package-years	13.3 (15)	12.2 (14)	14.0 (15)	0.047
Environmental tobacco smoke*	Yes	2023 (32.5%)	875 (31.4%)	1148 (33.3%)	0.121
Alcohol use*	Most of the days	1868 (30.0%)	891 (29.5%)	1047 (30.4%)	0.469
Diet	Percentage of energy from carbohydrates	68.1% (10)	68.4% (9)	67.9% (10)	0.050
	Percentage of energy from fat	17.2% (6)	17.4% (6)	17.0% (6)	0.002
	Percentage of energy from saturated fat	4.8% (2)	4.9% (2)	4.8% (2)	0.002
	Percentage of energy from protein	9.2% (1)	9.3% (1)	9.2% (2)	<0.001
Physical activity (METs)*	Sedentary or light active (<1.70)	4010 (67.9%)	1844 (71.3%)	2166 (65.2%)	<0.001
	Active or moderately active (1.70-1.99)	1580 (26.7%)	623 (24.1%)	957 (28.8%)	
	Vigorously active (>2)	318 (5.4%)	121 (4.7%)	197 (5.9%)	
**Fuel use**					
Main source of cooking fuel use*	Biomass	3456 (57.4%)	1445 (54.7%)	2011 (59.5%)	<0.001
Stove ventilation*	Not vented to the outside	1777 (28.5%)	876 (31.5%)	901 (26.2%)	<0.001
Main source of lighting fuel use*	Biomass	79 (1.3%)	32 (1.2%)	47 (1.4%)	0.524

*Missing values were 1 (<0.1%) for environmental tobacco smoke, and stove ventilation, 2 (<0.1%) for occupation, and education, 3 (<0.1%) for hypertension, smoking status, and lighting fuel use, 4 (<0.1%) for alcohol use, 13 (0.2%) for body-mass index, 15 (0.2%) for abdominal obesity, 180 (2.9%) for cholesterol, 188 (3%) for standard living index, 207 (3.3%) for main source of cooking fuel, 210 (3.4%) for triglycerides, and 321 (5.2%) for physical activity.

Data are mean (SD) or n (%).

DBP=diastolic blood pressure, HDL=high density lipoprotein, METs=metabolic equivalents; SBP=systolic blood pressure.

eTable 3. **Association of within-village variation in PM2.5 and biomass fuel use with mean and maximum carotid intima-media thickness in all participants and stratified by gender (**sensitivity analysis: mediators in the main model**).**

**Model**	**Exposure**	**All (n = 3278)**	**Men (n = 1693)**	**Women (n = 1585)**
**Outcome:** Mean CIMT (mm)		0.829 (0.25)	0.799 (0.26)	0.862 (0.24)
		Percent difference in CIMT (95% CI)	Percent difference in CIMT (95% CI)	Percent difference in CIMT (95% CI)
**Ambient Air Pollution**				
Model 3 (Full adjustment + HAP)*	PM 2.5 (1 μg/m^3)	1.79 (-0.31, 3.90)	2.98 (0.23, 5.72)	0.51 (-2.40, 3.43)
Model 4 (Model 3 + mediators)†	PM 2.5 (1 μg/m^3)	1.42 (-0.68, 3.52)	2.73 (-0.02, 5.47)	-0.16 (-3.08, 2.76)
**Household Air Pollution**				
Model 3 (Full adjustment + HAP)*	Biomass	1.60 (-0.46, 3.65)	1.77 (-0.89, 4.44)	
	Biomass (Vented)			-0.20 (-3.35, 2.95)
	Biomass (Not vented)			6.14 (1.40, 10.89)
Model 4 (Model 3 + mediators)†	Biomass	1.64 (-0.41, 3.69)	1.74 (-0.93, 4.41)	
	Biomass (Vented)			0.09 (-3.05, 3.23)
	Biomass (Not vented)			5.67 (0.93, 10.40)
**Outcome:** Maximum CIMT (mm)		0.865 (0.27)	0.833 (0.28)	0.899 (0.26)
**Ambient Air Pollution**				
Model 3 (Full adjustment + HAP)*	PM 2.5 (1 μg/m^3)	1.98 (-0.24, 4.20)	3.54 (0.65, 6.44)	0.24 (-2.85, 3.32)
Model 4 (Model 3 + mediators)†	PM 2.5 (1 μg/m^3)	1.59 (-0.62, 3.80)	3.28 (0.38, 6.17)	-0.50 (-3.58, 2.59)
**Household Air Pollution**				
Model 3 (Full adjustment + HAP)*	Biomass	1.62 (-0.57, 3.81)	1.73 (-1.08, 4.54)	
	Biomass (Vented)			-0.18 (-3.52, 3.16)
	Biomass (Not vented)			5.44 (0.42, 10.47)
Model 4 (Model 3 + mediators)†	Biomass	1.67 (-0.51, 3.86)	1.69 (-1.12, 4.50)	
	Biomass (Vented)			0.14 (-3.18, 3.46)
	Biomass (Not vented)			4.91 (-0.11, 9.93)

Analysis conducted in 10 multiple imputed datasets, using a linear mixed model accounting for within-between effects, with correction for selection bias through inverse probability weighting. CI = confidence interval; CIMT = carotid intima-media thickness; HAP = household air pollution; PM2.5 = particulate matter with an aerodynamic diameter of 2.5 micrometers or less.

* Model 3 was adjusted by age (modelled with natural spline, df=3), gender, occupation, education, standard living index, body-mass index, fruits and vegetables consumption, smoking status and environmental tobacco smoke, alcohol consumption, physical activity, biomass fuel use and whether stove was vented to the outside. The models for women did not include active smoking and have an interaction term between biomass fuel use and whether stove was vented to the outside.

† Model 4: Model3 + blood pressure, impaired fasting glucose and non-HDL cholesterol.

eTable 4. **Association of within-village variation in PM2.5 and biomass fuel use with mean and maximum carotid intima-media thickness in all participants and stratified by gender (**sensitivity analysis: multiple imputed data without correction for selection bias**).**

**Model**	**Exposure**	**All (n = 3278)**	**Men (n = 1693)**	**Women (n = 1585)**
**Outcome**: mean CIMT (mm)		0.829 (0.25)	0.799 (0.26)	0.862 (0.24)
		Percent difference in CIMT (95% CI)	Percent difference in CIMT (95% CI)	Percent difference in CIMT (95% CI)
**Ambient Air Pollution**				
Model 1 (basic adjustment)*	PM 2.5 (1 µg/m^3)	1.68 (-0.26, 3.63)	2.73 (0.07, 5.40)	0.59 (-2.13, 3.32)
Model 2 (full adjustment)†	PM 2.5 (1 µg/m^3)	1.54 (-0.39, 3.47)	2.63 (0.00, 5.27)	0.56 (-2.16, 3.27)
Model 3 (Model 2 + HAP)‡	PM 2.5 (1 µg/m^3)	1.52 (-0.41, 3.45)	2.56 (-0.08, 5.19)	0.52 (-2.19, 3.24)
**Household Air Pollution**				
Model 3 (Model 2 + HAP)‡	Biomass	1.36 (-0.56, 3.28)	2.17 (-0.44, 4.77)	
	Biomass (Vented)			-1.00 (-4.03, 2.02)
	Biomass (Not vented)			4.59 (-0.11, 9.30)
**Outcome**: maximum CIMT (mm)		0.865 (0.27)	0.833 (0.28)	0.899 (0.26)
**Ambient Air Pollution**				
Model 1 (basic adjustment)*	PM 2.5 (1 µg/m^3)	1.88 (-0.15, 3.92)	3.36 (0.58, 6.14)	0.42 (-2.44, 3.27)
Model 2 (full adjustment)†	PM 2.5 (1 µg/m^3)	1.74 (-0.29, 3.76)	3.23 (0.47, 6.00)	0.38 (-2.47, 3.23)
Model 3 (Model 2 + HAP)‡	PM 2.5 (1 µg/m^3)	1.72 (-0.31, 3.74)	3.17 (0.41, 5.94)	0.33 (-2.52, 3.18)
**Household Air Pollution**				
Model 3 (Model 2 + HAP)‡	Biomass	1.21 (-0.81, 3.23)	1.94 (-0.79, 4.67)	
	Biomass (Vented)			-1.09 (-4.28, 2.11)
	Biomass (Not vented)			4.01 (-0.96, 8.98)

Analysis conducted in 10 multiple imputed datasets, using a linear mixed model accounting for within-between effects, without correction for selection bias. CI = confidence interval; CIMT = carotid intima-media thickness; HAP = household air pollution; PM2.5 = particulate matter with an aerodynamic diameter of 2.5 micrometers or less.

* Model 1 was adjusted by age (modelled with natural spline, df=3) and gender.

† Model 2 was additionally adjusted by occupation, education, standard living index, body-mass index, fruits and vegetables consumption, smoking status and environmental tobacco smoke, alcohol consumption, and physical activity. The models for women did not include active smoking.

‡ Model 3 was additionally adjusted by biomass fuel use and whether stove was vented to the outside. The models for women have an interaction term between biomass fuel use and whether stove was vented to the outside.

eTable 5. **Association of within-village variation in PM2.5 and biomass fuel use with mean and maximum carotid intima-media thickness in all participants and stratified by gender (**sensitivity analysis: complete case analysis, with and without correction for selection bias**).**

**Model**	**Exposure**	**All (n = 3074)**	**Men (n = 1597)**	**Women (n = 1477)**
**Outcome**: mean CIMT (mm)		0.828 (0.25)	0.797 (0.26)	0.861 (0.24)
		Percent difference in CIMT (95% CI)	Percent difference in CIMT (95% CI)	Percent difference in CIMT (95% CI)
**Ambient Air Pollution**				
Model 3 (Model 2 + HAP)*, with selection bias correction	PM 2.5 (1 μg/m^3)	1.70 (-0.43, 3.83)	2.91 (0.17, 5.65)	0.55 (-2.40, 3.50)
Model 3 (Model 2 + HAP)*, without selection bias correction	PM 2.5 (1 μg/m^3)	1.42 (-0.54, 3.38)	2.41 (-0.24, 5.06)	0.56 (-2.20, 3.31)
**Household Air Pollution**				
Model 3 (Model 2 + HAP)*, with selection bias correction	Biomass	1.77 (-0.26, 3.80)	1.52 (-1.13, 4.18)	
	Biomass (Vented)			0.43 (-2.81, 3.67)
	Biomass (Not vented)			6.71 (1.80, 11.63)
Model 3 (Model 2 + HAP)*, without selection bias correction	Biomass	1.64 (-0.31, 3.58)	2.06 (-0.56, 4.68)	
	Biomass (Vented)			-0.57 (-3.70, 2.56)
	Biomass (Not vented)			5.56 (0.70, 10.42)
**Outcome**: maximum CIMT (mm)		0.863 (0.27)	0.831 (0.28)	0.898 (0.26)
**Ambient Air Pollution**				
Model 3 (Model 2 + HAP)*, with selection bias correction	PM 2.5 (1 μg/m^3)	1.92 (-0.32, 4.16)	3.58 (0.69, 6.47)	0.28 (-2.82, 3.38)
Model 3 (Model 2 + HAP)*, without selection bias correction	PM 2.5 (1 μg/m^3)	1.65 (-0.41, 3.71)	3.11 (0.33, 5.90)	0.35 (-2.53, 3.23)
**Household Air Pollution**				
Model 3 (Model 2 + HAP)*, with selection bias correction	Biomass	1.81 (-0.32, 3.94)	1.48 (-1.31, 4.28)	
	Biomass (Vented)			0.48 (-2.94, 3.89)
	Biomass (Not vented)			6.05 (0.86, 11.23)
Model 3 (Model 2 + HAP)*, without selection bias correction	Biomass	1.51 (-0.53, 3.55)	1.88 (-0.86, 4.63)	
	Biomass (Vented)			-0.69 (-3.97, 2.60)
	Biomass (Not vented)			4.95 (-0.15, 10.05)

Analysis conducted in complete case data, using a linear mixed model accounting for within-between effects, with and without correction for selection bias through inverse probability weighting. CI = confidence interval; CIMT = carotid intima-media thickness; HAP = household air pollution; PM2.5 = particulate matter with an aerodynamic diameter of 2.5 micrometers or less.

* Model 3 was adjusted by age (modelled with natural spline, df=3), gender, occupation, education, standard living index, body-mass index, fruits and vegetables consumption, smoking status and environmental tobacco smoke, alcohol consumption, physical activity, biomass fuel use and whether stove was vented to the outside. The models for women did not include active smoking and have an interaction term between biomass fuel use and whether stove was vented to the outside.

References

1. Kinra S, Bowen LJ, Lyngdoh T, et al. Sociodemographic patterning of non-communicable disease risk factors in rural India: a cross sectional study. *BMJ*. 2010;341(sep27 1):c4974-c4974. doi:10.1136/bmj.c4974.

2. Kinra S, Radha Krishna K V., Kuper H, et al. Cohort profile: Andhra Pradesh children and parents study (APCAPS). *Int J Epidemiol*. 2014;43(5):1417-1424. doi:10.1093/ije/dyt128.

3. Alberti KGMM, Eckel RH, Grundy SM, et al. Harmonizing the metabolic syndrome: A joint interim statement of the international diabetes federation task force on epidemiology and prevention; National heart, lung, and blood institute; American heart association; World heart federation; International . *Circulation*. 2009;120(16):1640-1645. doi:10.1161/CIRCULATIONAHA.109.192644.

4. Bowen L, Bharathi AV, Kinra S, Destavola B, Ness A, Ebrahim S. Development and evaluation of a semi-quantitative food frequency questionnaire for use in Urban and rural India. *Asia Pac J Clin Nutr*. 2012;21(3):355-360.

5. Matsuzaki M, Sullivan R, Ekelund U, et al. Development and evaluation of the Andhra Pradesh Children and Parent Study Physical Activity Questionnaire (APCAPS-PAQ): a cross-sectional study. *BMC Public Health*. 2016;16(1):48. doi:10.1186/s12889-016-2706-9.

6. Seaman SR, White IR. Review of inverse probability weighting for dealing with missing data. *Stat Methods Med Res*. 2013;22(3):278-295. doi:10.1177/0962280210395740.

7. Robins JM, Hernan MA, Brumback B. Marginal structural models and causal inference in epidemiology. *Epidemiology*. 2000;11(5):550-560.

8. Seaman SR, White IR, Copas AJ, Li L. Combining Multiple Imputation and Inverse-Probability Weighting. *Biometrics*. 2012;68(1):129-137. doi:10.1111/j.1541-0420.2011.01666.x.

9. Sterne JA, White IR, Carlin JB, et al. Multiple imputation for missing data in epidemiological and clinical research: potential and pitfalls. *BMJ*. 2009;338:b2393. doi:10.1136/bmj.b2393.

10. Buuren S van, Groothuis-Oudshoorn K. **mice** : Multivariate Imputation by Chained Equations in *R*. *J Stat Softw*. 2011;45(3). doi:10.18637/jss.v045.i03.

11. Little RJA. Missing-data adjustments in large surveys. *J Bus Econ Stat*. 1988;6(3):287-296. doi:10.1080/07350015.1988.10509663.

12. Vink G, Lazendic G, Buuren S Van. Partitioned predictive mean matching as a multilevel imputation technique. *Psychol Test Assess Model*. 2015;5(4):1-16.

13. R Core Team. R. *R Core Team*. 2017. doi:3-900051-14-3.

14. Wickham H. *Tidyverse: Easily Install and Load "Tidyverse" Packages.*; 2016. https://cran.r-project.org/package=tidyverse.

15. Robitzsch A, Grund S, Henke T. miceadds: Some additional multiple imputation functions, especially for mice. 2017. https://cran.r-project.org/package=miceadds.

16. Bates D, Machler M, Bolker BM, Walker SC. Fitting Linear Mixed-Effects Models using lme4. *J Stat Softw*. 2015;67(1):1-48. doi:10.18637/jss.v067.i01.

17. Bates DM. *Lme4: Mixed-Effects Modeling with R*. Springer; 2010. http://lme4.r-forge.r-project.org/book/.

18. Viechtbauer W. Conducting Meta-Analyses in R with the metafor Package. *J Stat Softw*. 2010;36(3):1-48. doi:10.1103/PhysRevB.91.121108.

19. Wickham H. ggplot2. *Wiley Interdiscip Rev Comput Stat*. 2011;3(2):180-185. doi:10.1002/wics.147.

20. Gordon M, Lumley T. forestplot: Advanced Forest Plot Using "grid" Graphics. 2017. https://cran.r-project.org/package=forestplot.
